# Characterization of the Distal Polyadenylation Site of the ß-Adducin (Add2) Pre-mRNA

**DOI:** 10.1371/journal.pone.0058879

**Published:** 2013-03-15

**Authors:** Luisa Costessi, Fabiola Porro, Alessandra Iaconcig, Mirjana Nedeljkovic, Andrés Fernando Muro

**Affiliations:** International Centre for Genetic Engineering and Biotechnology (ICGEB), Trieste, Italy; CNRS UMR7275, France

## Abstract

Most genes have multiple polyadenylation sites (PAS), which are often selected in a tissue-specific manner, altering protein products and affecting mRNA stability, subcellular localization and/or translability. Here we studied the polyadenylation mechanisms associated to the beta-adducin gene (Add2). We have previously shown that the Add2 gene has a very tight regulation of alternative polyadenylation, using proximal PAS in erythroid tissues, and a distal one in brain. Using chimeric minigenes and cell transfections we identified the core elements responsible for polyadenylation at the distal PAS. Deletion of either the hexanucleotide motif (Hm) or the downstream element (DSE) resulted in reduction of mature mRNA levels and activation of cryptic PAS, suggesting an important role for the DSE in polyadenylation of the distal Add2 PAS. Point mutation of the UG repeats present in the DSE, located immediately after the cleavage site, resulted in a reduction of processed mRNA and in the activation of the same cryptic site. RNA-EMSA showed that this region is active in forming RNA-protein complexes. Competition experiments showed that RNA lacking the DSE was not able to compete the RNA-protein complexes, supporting the hypothesis of an essential important role for the DSE. Next, using a RNA-pull down approach we identified some of the proteins bound to the DSE. Among these proteins we found PTB, TDP-43, FBP1 and FBP2, nucleolin, RNA helicase A and vigilin. All these proteins have a role in RNA metabolism, but only PTB has a reported function in polyadenylation. Additional experiments are needed to determine the precise functional role of these proteins in Add2 polyadenylation.

## Introduction

Most eukaryotic mRNAs acquire an untemplated polyA tail at their 3′ end by a process named polyadenylation. The addition of the polyA tail is the result of a highly coordinated two-step process, in which the pre-mRNA is first cleaved at the CA dinucleotide of the cleavage site (CS) and then the polyA tail is subsequently added. This process requires the formation of protein-RNA complexes at the core polyadenylation elements: the hexanucleotide motif (Hm, AAUAAA or a variant, located 20–30 nt upstream of the CS), and the U/UG-rich element located downstream of the CS (downstream element, DSE). The Hm is the binding site of the cleavage and polyadenylation specificity factor (CPSF) complex, while the DSE is bound by the cleavage stimulation factor (CstF). Recent genome- and transcriptome-wide analyses suggest that the consensus motif of CstF-64 corresponds to UGUGU [1], [2]. Both the Hm and the DSE are critical for correct recognition and efficient processing of the PAS. The presence of auxiliary upstream elements (USE) and downstream sequences, recognized by specific trans-acting factors, are known to modulate polyadenylation efficiency [3], [4], [5], [6], [7], [8], [9], [10], [11], [12]. Many non-canonical Hm rely on the presence of potent DSE to recruit the basal polyadenylation machinery [13].

Interestingly, about 70% of mammalian genes contain multiple polyadenylation sites (PAS), generating transcripts differing in the 3′UTR or coding regions [14]. Usage of one PAS over another is attributed to a balance between the relative strength of the core elements, and the presence of additional auxiliary sequences, factors and/or fine regulation of the levels of specific components of the polyadenylation machinery. Alternative polyadenylation (APA) is an important mechanism of gene regulation since the length of the 3′UTR affects mRNA fate. Depending on the 3′ UTR length, transcript isoforms can contain different combinations of cis acting elements, which may affect subcellular localization, translation efficiency and/or stability [15], [16]. The beta adducin (Add2) mRNA is a clear example of APA occurring in a tissue-specific manner [17]. The Add2 gene is transcribed from two tightly controlled tissue-specific promoters, one used in erythroid tissues and another one in neuronal ones. The AUG and stop codons are encoded by common exons, thus generating the same ORF in both tissues [17]. The Add2 terminal exon contains tandem polyadenylation sites (PAS): two proximal PAS mainly used in erythroid tissues (A1 PAS and A2-3 PAS), and one distal PAS exclusively used in brain (A4 PAS) [17]. In brain, the Add2 mRNAs contain an unusually long 3′UTR of 5–6 kb, depending on the species, and are localized in dendrites [17], [18].

Adducins are membrane skeletal proteins encoded in mammalian genomes by three closely related genes: α-, ß-, and γ-adducins (Add1, Add2 and Add3, respectively). Adducins are present in the membrane skeleton of cells as homo- and heterodimers where they cap the fast growing end of actin filaments [19]. While Add1 and Add3 are expressed ubiquitously, Add2 is restricted to erythroid tissues and the nervous system [20], . In the brain, the adducin heterodimer is present in growth cones of axons, presynaptic nerve terminals and post-synaptic dendritic spines [23], [24]. It is very abundant in hippocampal dendritic spines. Add2 participates in the assembly of synapses, controls synaptic plasticity and their stability [25], [26], and its genetic inactivation in mice leads to LTP, LTD, learning and motor-coordination deficits [18], [27].

We have previously predicted the putative core polyadenylation elements of the brain-specific PAS [17], but the determination of their functionality, the protein factors recognizing the Add2 PAS pre-mRNA and the mechanisms regulating polyadenylation were still lacking. To address these questions, we generated chimeric minigenes containing the polyadenylation regions of Add2, and accurately reproduced the use of the distal A4 PAS by transfecting those constructs into HeLa and HEK293 cells. We have shown here the essential roles of the Hm and DSE for the correct processing of the chimeric transcript. Deletion of either the Hm or DSE resulted in the activation of cryptic PASs located ∼200 bases upstream. Using RNA-EMSA, we have seen that protein complexes recognize the distal PAS of Add2. Pull-down experiments allowed us to identify a set of proteins recognizing the USE and DSE of the distal Add2 PAS, some of them with not yet reported role in polyadenylation. The information presented in this manuscript is the first step to understand the molecular mechanisms at the basis of alternative polyadenylation of the ß-adducin pre-mRNA.

## Materials and Methods

### Construction of Minigene Constructs

**Figure 1:**
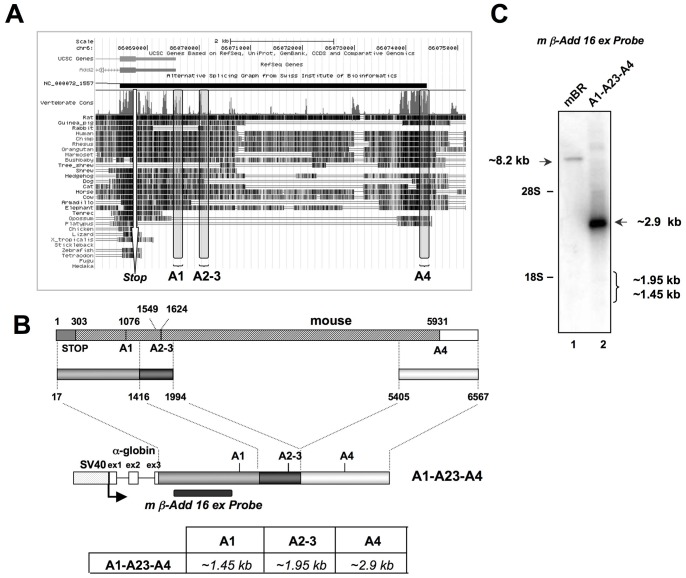
The minigene-encoded transcript is cleaved and polyadenylated at the distal PAS. **Panel A:** Scheme showing the homology among vertebrates in the last exon of the Add2 gene (UCSC browser). The A1, A23 and A4 PAS are indicated. The stop codon is indicated (“Stop”). **Panel B:** Scheme of the last exon of the mouse Add2 gene (top). The regions used for the generation of the A1-A23-A4 construct. The numbering refers to the first base of the last Add2 exon. The probe used in the Northern blots experiments is indicated. The expected size of the transcripts originating from the different PAS is shown. **Panel C:** Northern blot experiment of RNA prepared from HeLa cells transfected with the A1-A23-A4 construct. The position of the Add2 brain mRNA (lane 1), expected plasmid encoded transcripts (lane 2) and rRNA are indicated. Mouse brain RNA (mBR) was used as control.

The minigenes used for the transfection experiments were generated by inserting the fragments containing the tissue-specific polyadenylation sites of ß-adducin and their flanking sequences (A1, A23 and A4 inserts, Figure 1B) into the pBS SV40- αGlo-linker construct. The pBS SV40- αGlo-linker construct was prepared by replacing the last 344 bp of exon 3 (including the α-Globin PAS) with an ad-hoc polylinker, of an ampicillin resistant version of the pSV-α1 plasmid [28] which contained the SV40 promoter sequence (352 bp) and the genomic sequence of human α-Globin gene [29]. The different fragments containing the tissue-specific polyadenylation sites of ß-adducin and their flanking sequences (A1, A23 and A4 inserts, Figure 1B) were obtained by PCR from mouse genomic DNA and the absence of mutation was confirmed by sequencing. The final chimeric minigenes were used to transfect HeLa and HEK293 cells.

### *In vitro* Site Directed Mutagenesis

The plasmid containing the target sequence (50 ng) and the oligonucleotides primers containing the desiderate mutations (complementary to opposite strands of the target sequence) were added to the reaction mix (2.5 U/µl *PfuTurbo* DNA polymerase (Stratagene), 1X reaction buffer, 200 µM dNTP mix). An extension reaction was performed and the amplification product was digested with DpnI to eliminate the parental DNA template allowing the selection of mutation-containing synthesized DNA. The DpnI-resistant DNA was then transformed into competent bacterial cells. This method was used to create the deletions and mutations constructs of ß-adducin chimeric minigenes.

### Cell Culture Transient Transfections

Liposome-mediated transfections of plasmid DNA into HeLa and HEK293 cell lines were performed using Lipofectamine 2000 transfection reagent (Invitrogen) according to the manufacturer’s instructions. Along with the test minigene construct, each plasmid DNA mix contained the plasmid expressing SV40 T-antigen to stimulate SV40 promoter activity. pEGFP-C2 (Clontech) was used as transfection efficiency control. The plasmid DNA used for transfections was purified with JetStar columns (Genomed).

### RNA Preparation, Synthesis of cDNA, PCRs

Total cellular RNA was isolated with TRI REAGENT (Sigma) following manufacturer’s instructions. cDNA was prepared from total RNA (1–2 µg in a final volume of 20 µl) by using M-MLV reverse transcriptase (RT) (Invitrogen) following manufacturer’s protocols.

The polymerase chain reaction (PCR) was performed as described by the Taq polymerase manufacturers (Roche or NEB). The oligonucleotide primers used to perform the different amplifications are shown below.

*3′-RACE-PCR.* After the cDNA synthesis using an oligo dT primer containing an anchor sequence at the 5′ end, a first PCR was performed using as forward primer an internal oligonucleotide complementary to the cDNA and a primer complementary to the anchor sequence (3′RACE 1^st^ reaction). To give further specificity to the reaction a second couple of primers was used to amplify the first PCR product (3′RACE 2^nd^ reaction). 3′RACE PCR products were agarose gel purified and sequenced (Macrogen).

### Northern Blot Analysis

The Northern Blot analysis was performed as described [17]. Briefly, total RNA (2–10 µg) was loaded in 1.5% agarose formaldehyde gels, transferred to nylon membranes, and hybridized in ULTRAhyb® Ultrasensitive Hybridization Buffer (Ambion) with the appropriate DNA probe labeled with (α-^32^P) dCTP. The DNA fragments used as probes were obtained by PCR from genomic DNA (mß-Add 16 ex Probe) and pEGFP-C2 vector (GFP Probe), labeled with Rediprime II Random Prime Labeling System (Amersham Bioscience) and then purified from unincorporated nucleotides by Nick columns (Amersham bioscience).

### *In vitro* Transcription

For RNA-EMSA and RNA-pull down, RNA was in vitro synthesized with T7 RNA polymerase (Stratagene) from a DNA template (PCR amplified) containing the T7 RNA polymerase promoter sequence (oligonucleotides are indicated below) as described by the manufacturer. In order to radioactively label RNAs (hot RNAs), (α-^32^P) UTP was added to the transcription mix. The cold reaction was phenol-chloroform purified while the hot one through nick-columns (Amersham bioscience). Denaturing agarose (cold RNA) or polyacrylamide gels (hot RNA) were run to check quality and size of the synthesized RNA.

### RNA-EMSA

RNA-EMSAs were conducted according to procedures already described (Buratti 2011) with minor modifications. Radioactively labeled RNA fragments were incubated in EMSA binding buffer (5.2 mM Hepes pH 7.9, 1 mM MgCl_2_, 0.8 mM MgAc, 0.52 mM DTT, 3.8% glycerol, 0.75 mM ATP, 1 mM GTP, heparin 5–15 µg/µl) with HeLa nuclear extract (Cilbiotech). To modulate the stringency of the binding we used different final concentrations of heparin (5 µg/µl and 15 µg/µl) and nuclear extract (50 µg and 2 µg), as described in the Legends to the Figures. The RNA-proteins mix was incubated at room temperature for 20 minutes before running on native polyacrylamide gels (5%). When cold RNA fragments were used as competitors, a pre-incubation between cold RNA and the nuclear extract was done before adding the radiolabeled RNA. Gels were dried before X-OMAT film or Cyclone (Packard) exposure.

### RNA-Pull Down, SDS-PAGE, MS and Western Blot

RNA-Pull down analysis was performed as described with small modifications (Buratti 2001). Cold RNAs (50 µg)-beads were incubated with 0.8 mg of HeLa cell nuclear (Cilbiotech). After the washing steps, proteins bound to RNA-beads were separated on a gradient SDS–PAGE gel (gradient 8–15% or 10%). After running, gels were either stained with colloidal Coomassie G-250 or transferred to nitrocellulose membrane for western blot analysis. Sequence analysis from the Coomassie-stained bands excised from the SDS–PAGE gel was performed by Protein Network Group from ICGEB, Trieste, Italy.

Western blot analyses were performed as previously described (Porro 2010). For western blot the following primary antibodies were used: anti TDP-43 (ProteinTech), PTB (antibody kindly provided by Dr. Buratti, ICGEB, Trieste, Italy). The results were quantified with Quantity One software (Bio-Rad Laboratories).

### Sequences of Primers and RNAs

Primers: m ß-Add 16 ex dir 1 (5′ AGGAGGAGGAGCCGAGCGTA 3′) and m ß-Add 16 ex rev 1 (5′ TCCTCCTTCCCATTCACCAC 3′) for synthesis of the mß-Add16 ex Probe; GFP813FW (5′ CGGCGTGCAGTGCTTCAGCCGCTAC 3′) and GFPendRV (5′ CTTGTACAGCTCGTCCATGCCGAGAG 3′) for the GFP Probe; BR A4 dir 1 tail (5′ CGGCGTGCAGTGCTTCAGCCGCTAC 3′), dT-anchor 1 (5′ TAGGAATTCTCGAGCGGCCGCT(17)A/C/G 3′), mXhoI dir1 (5′ CCGCTCGAGTCTGCCTTTCTCTATGCTA 3′) and anchor 1 (5′ CCGCTCGAGTCTGCCTTTCTCTATGCTA 3′) for the 3′ RACE reactions; T7 mBRA4 dir 1 (5′ TAATACGACTCACTATAGGTGTTTGGGGTGGACTCTG 3′) and mBR A4 rev a (5′ CTCCCACCTCCCCAGGAG 3′) for the generation of the DNA template for T7-directed in vitro transcription.

## Results

### Identification of the Elements Participating in Add2 Pre-mRNA Polyadenylation

**Figure 2:**
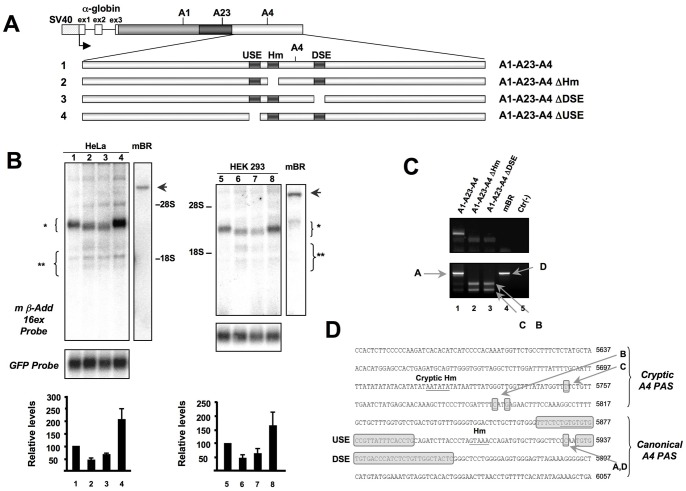
Deletion of the Hm or DSE results in activation of cryptic PAS. **Panel A:** Scheme of the minigenes containing deletions of the core polyadenylation elements of the A4 PAS. **Panel B:** Northern blot analysis of RNA prepared from HeLa (Lanes 1–4) and HEK293 (Lanes 5–8) cells transfected with the constructs of Panel A. Add2 transcripts were detected with the mß-Add 16ex probe. A plasmid encoding for the GFP was used to normalize for differences in transfection efficiency. The relative levels (mean±SD of 3 different experiments, Add2/GFP ratio) are shown in the bar graph (bottom). The position of the expected Add transcripts (*, ∼2.75–2.9 kb; **, 1.95–1.45 kb) and rRNAs (28S and 18S) are indicated. The arrow indicates the position of the Add2 mRNA of mouse brain (∼8.2 kb). **Panel C:** RACE-PCR experiment of the HeLa cells transfection to map the cleavage site of Add2 transcripts (see Materials and Methods section). The top panel corresponds to the first PCR reaction. The bottom panel corresponds to the semi-nested PCR reaction. The Add2 polyadenylation sites are indicated: “A” and “D”, for the plasmid or brain mRNAs, “B” and “C” for the cryptic sites. Identical results were obtained in HEK293 cells. **Panel D:** Nucleotide sequence of the A4 region. The canonical and cryptic PAS, USE, Hm, CS and DSE are indicated. The large boxes correspond to the deletions introduced in the constructs used above.

An effective strategy to study the mechanisms of pre-mRNA processing is based on the use of chimeric reporter minigenes containing the genomic regions of interest [28]. Expression of minigene products is then tested by Northern blot analysis or RT-PCR of total RNA prepared from transfected cells. In order to study the molecular mechanisms of polyadenylation of the mouse Add2 gene, we constructed a chimeric minigene by replacing the alpha-globin natural PAS in an ampicillin-resistant version of the pSV-α1 plasmid [29], [30] by the three reported Add2 PASs (named A1, A23 and A4) and their respective flanking regions [17]. These regions showed to be highly conserved among species, supporting the idea that the most important regulatory elements of polyadenylation reside within those regions (Figure 1A and Figures S1, S2 and S3). The generated A1-A23-A4 construct reproduced the natural gene structure of the mouse *Add2* last exon and downstream region (Figure 1A and 1B). To attain high levels of expression the minigene was under the transcriptional control of the SV40 promoter region. Northern blot analysis of total RNA prepared from HeLa cells transfected with the A1-A23-A4 construct resulted in the appearance of a single band, which corresponded with the expected mRNA species using the distal A4 PAS (2.9 kb w/o polyA tail, Figure 1B and 1C). Cleavage and polyadenylation at the A4 PAS was confirmed by RACE-PCR, which showed that the selected site was the natural one used by the endogenous Add2 mRNA in neuronal tissues (Figure 2C, Lanes 1 and 4 and Figure 2D). A RACE-PCR approach using primers annealing to the proximal PAS region detected very small amounts of mRNAs corresponding to the use of the proximal PASs (not shown). These experiments demonstrated that, when transfected into HeLa cells, the construct containing the different polyadenylation regions of the Add2 gene accurately reproduced the pattern observed in brain by the Add2 gene, where the A4 is the major PAS used [17].

In order to identify the cis-acting regulatory elements present in the distal A4 PAS, we aligned the mouse Add2 sequence with those of 24 vertebrates and performed a manual inspection of the sequence ([17] and Figure S1). We identified the putative polyadenylation core elements: the hexanucleotide motif (Hm), upstream and downstream sequence element (USE and DSE, respectively). These elements showed to be highly conserved in the Add2 gene among vertebrates (Figure 1A and Figure S1) [17]. The conservation dropped immediately downstream of the DSE, as already observed in many other genes [31], suggesting that all elements involved in polyadenylation were located around the CS. On the contrary, conservation among vertebrates of the proximal PASs was more limited, with no obvious core elements in the A23 PAS (Figure 1A and Figures S2 and S3) [17].

To test their functionality, each of the putative polyadenylation core elements of the A4 PAS was deleted from the original A1-A23-A4 minigene (Figure 2A and Figure S4) to generate the A1-A23-A4 ΔUSE, A1-A23-A4 ΔHm and A1-A23-A4 ΔDSE constructs, containing deletions of the USE, Hm and DSE, respectively. This set of constructs was transfected into HeLa cells and the mRNA detected by Northern blot analysis. We observed that absence of either the Hm or DSE resulted in a reduction in the amount and size of the chimeric α-globin-Add2 mRNAs, while an increase in the mRNA levels was observed when the USE was deleted (Figures 2B, lanes 1–4). 3′RACE and subsequent sequencing analysis of the PCR products showed that the differences in size of the mRNA generated by the A1-A23-A4 ΔH and A1-A23-A4 ΔDSE constructs were due to the activation of two cryptic PASs, located about 200 bases upstream of the natural A4 PAS (Figure 2C and 2D). Similar results were obtained after transfecting the same constructs into HEK293 cells (Figure 2B, lanes 5–8), suggesting that the observed differences were not due to cell-specific effects. Therefore, these experiments confirmed the importance of the Hm and DSE for the definition and correct 3′end processing of the Add2 pre-mRNA at the A4 PAS.

### Mutation of the GU Repeats of the A4 DSE Reduces Pre-mRNA Processing and Activates Two Cryptic PASs

**Figure 3:**
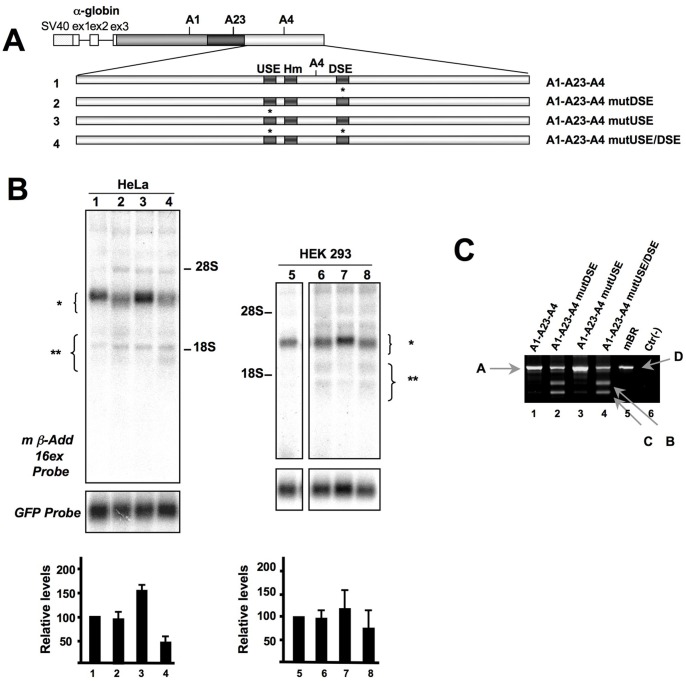
Point mutations in the DSE also result in the activation of cryptic PAS. **Panel A:** Scheme of the constructs used in Panel B. The DSE and USE were mutated (indicated by an asterisk) as shown in detail in the Materials and Methods section. **Panel B:** Northern blot of RNA prepared from HeLa (lanes 1–4) and HEK293 (lanes 5–8) cells transfected with the constructs shown in Panel A, hybridized with the mß-Add 16ex (top panel) or the GFP probes (bottom panel). The position of the rRNAs is indicated, as well as the position of the expected RNA bands (indicated by brackets). The bar graph indicates the relative levels of expression normalized with the GFP signal (Add2/GFP ratio, mean±SD of three different experiments). **Panel C:** RACE-PCR experiment of the HeLa cells transfection to map the cleavage site of Add2 transcripts. Only the second semi-nested PCR reaction is shown. The Add2 polyadenylation sites are indicated: “A” and “D”, for the plasmid or brain mRNAs, “B” and “C” for the cryptic sites. Identical results were obtained in HEK293 cells.

To more finely map the USE and DSE we performed point mutations to the elements (see Material and Methods Section). We generated the A1-A23-A4 mutDSE, A1-A23-A4 mutUSE and A1-A23-A4 mutUSE/DSE constructs which contained point mutations in the GU repeats of the Add2 USE, DSE and a combination of both sites mutated, respectively (see Figure S4). When these constructs were transfected into HeLa cells, we observed that mutation of the DSE resulted in the generation of mRNAs cleaved at both the natural A4 PAS and at the cryptic sites, as determined by RACE-PCR of the 3′ end (Figure 3B, lane 2 and Figure 3C). A very small amount of mRNA polyadenylated at the proximal A1 and A23 PAS was also detected. In a similar way to what observed after the deletion of the USE, mutation of the UG repeats present in the USE resulted in the increase of mRNA levels (Figure 3B, Lane 3). When both sites were mutated we observed a polyadenylation pattern similar to that of the DSE mutation alone (Figure 3B, lane 4). Similar results were obtained after transfecting HEK293 cells (Figure 3B, lanes 5–8).

### Optimization of the Hexanucleotide Motif Results in an Increase in Add2 mRNA

**Figure 4:**
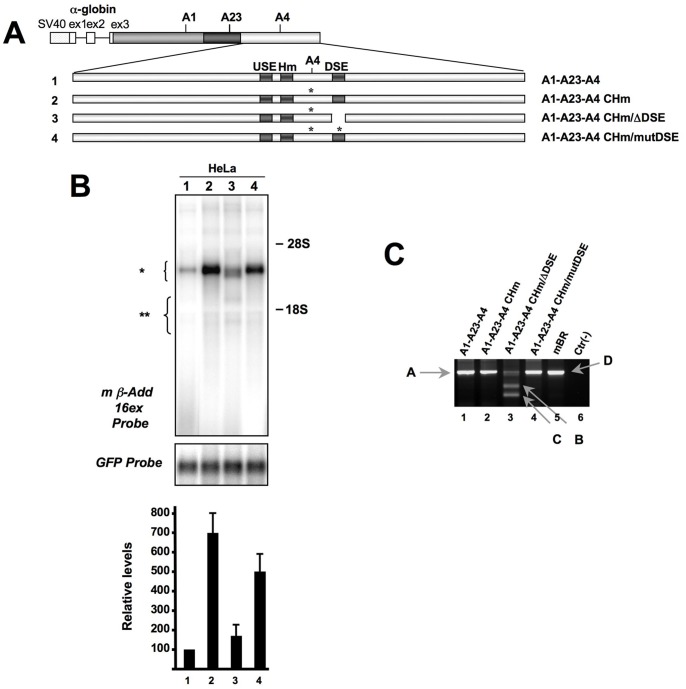
The Add2 A4 PAS DSE is necessary even after reversion of the AGUAAA to AAUAAA. **Panel A:** Scheme of the constructs used in Panel B. The hexanucleotide motif AGUAAA was optimized to the canonical AAUAAA sequence (A1-A23-A4 CHm construct). The constructs having the optimized Hm together with a deletion and point mutations in the DSE are shown (A1-A23-A4 CHm/ΔDSE and A1-A23-A4 CHm/mutDSE, respectively). **Panel B:** Northern blot of RNA prepared from HeLa (lanes 1–4) cells transfected with the constructs shown in Panel A, hybridized with the mß-Add 16ex (top panel) or the GFP probes (bottom panel). The position of the rRNAs is indicated, as well as the position of the expected RNA bands (indicated by brackets). The bar graph indicates the relative levels of expression normalized with the GFP signal (Add2/GFP ratio, mean±SD of three different experiments). **Panel C:** RACE-PCR experiment of the HeLa cells transfection to map the cleavage site of Add2 transcripts. Only the second semi-nested PCR reaction is shown. The Add2 polyadenylation sites are indicated: “A” and “D”, for the plasmid or brain mRNAs, “B” and “C” for the cryptic sites.

To determine the role of the non-canonical hexamer AGUAAA and to elucidate the interplay of the canonical hexamer with the Add2 A4 PAS DSE we reverted the AGUAAA sequence to a canonical AAUAAA hexamer. Therefore, we constructed an additional series of plasmids containing the optimized AAUAAA PAS (canonical hexanucleotide motif, CHm) in combination with the natural Add2 DSE (A1-A23-A4 CHm), a deletion of the DSE (A1-A23-A4 CHm/ΔDSE) or point mutations in the GU repeats of the Add2 DSE (A1-A23-A4 CHm/mutDSE) (Figure 4A). After transfection of these constructs into HeLa cells and Northern blot analysis, we observed that a single-base mutation in the hexanucleotide motif resulted in a 6–7 fold increase in the amount of mRNA, confirming published data that the canonical hexamer was more efficient than non-canonical hexanucleotide motifs, such as the AGUAAA present in the Add2 A4 PAS (Figure 4B, Lanes 1 and 2). However, we observed that the efficiency of the optimized PAS was highly dependent on the presence of the natural DSE, since its deletion resulted in an important reduction of the amount of Add2 mRNA and in the activation of the same cryptic sites described above (Figures 4B and 4C, Lane 3). Mutation of the GU repeats present in the DSE also resulted in a reduction in the mRNA levels, although the effect was considerably less marked than the complete DSE deletion (Figure 4B, Lane 4).

### Characterization of the Trans-acting Factors Binding to the A4 PAS of the Add2 Pre-mRNA

**Figure 5:**
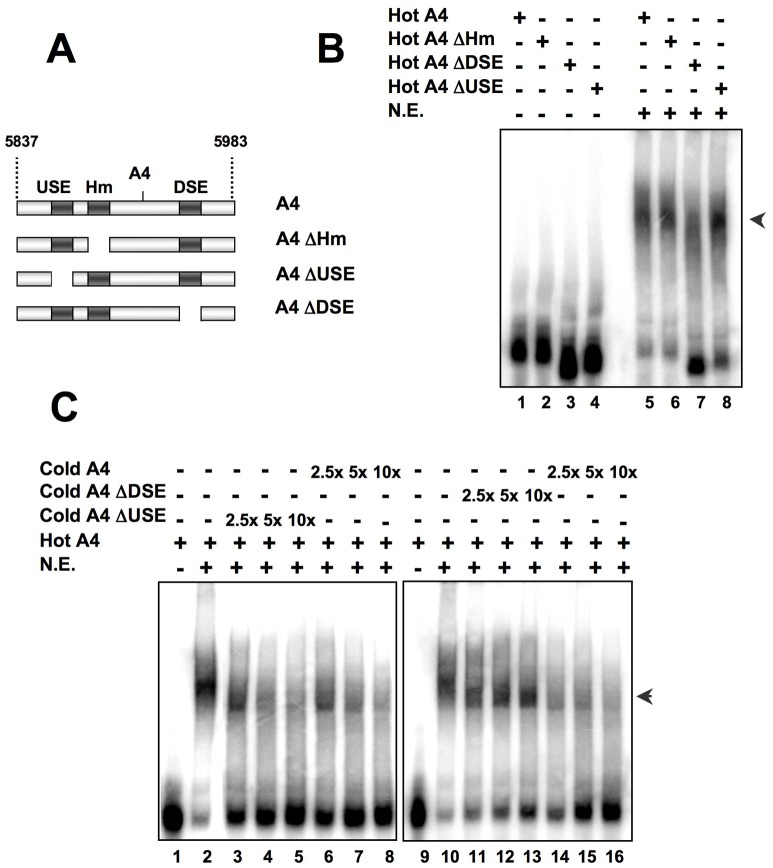
The A4 PAS region forms RNA-protein complexes. **Panel A:** Scheme of the RNA probes and competitors used in Panels B, C and D (see Figure S4 for the complete sequence). **Panel B:** Hot RNAs (A4, ΔHm, ΔDSE and ΔUSE) were incubated with 50 µg of HeLa nuclear extracts and 5 µg/µl of heparin (lanes 5–8), complexes separated in a polyacrylamide gel and autoradiographed. The arrow indicates the RNA-protein complexes. **Panel C:** The complexes formed in Panel B with radioactive A4 RNA were competed with different cold RNAs (cold A4, lanes 6–8 and 14–16; cold ΔDSE, lanes 11–13; cold ΔUSE, lanes 3–5). Lanes 1 and 9 corresponds to free probe (no nuclear extract).

We then performed EMSA with HeLa nuclear protein extracts and radioactive RNA probes of the wt or deleted A4 PAS (Figure 5). All probes were able to form RNA-protein complexes with nuclear protein extracts (Figure 5B, indicated by an arrow), but less amount of RNA-protein complex was formed with the ΔDSE probe (Figure 5B Lane 7), while the amount of complex formed by the ΔUSE probe was higher (Figure 5B, Lane 8), roughly correlating with the levels of mRNA detected in the Northern blot analysis shown in Figure 2B. Competition experiments confirmed the specificity of the complexes and the important contribution of the DSE, as those ones formed with the wt probe were competed out both by cold wt RNA and cold ΔUSE RNA, but not by cold ΔDSE RNA competitor (Figure 5C). These results underscore the essential role of the DSE in RNA-protein complex formation and in cleavage and polyadenylation of the A4 PAS.

**Figure 6:**
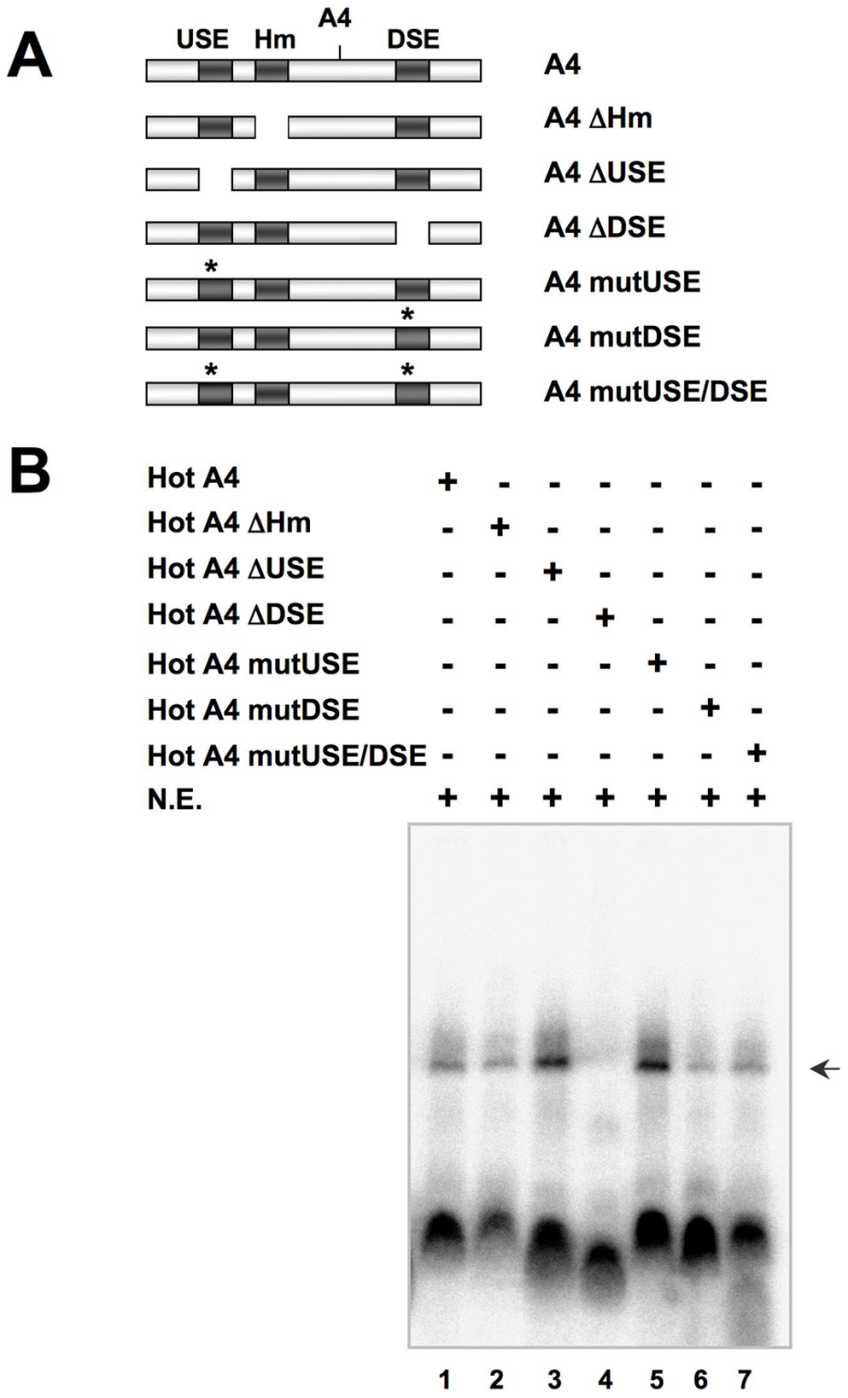
An RNA lacking the DSE is not able to form RNA-protein complexes under more stringent incubation conditions. **Panel A:** Scheme of the RNA probes and competitors used in Panel B. Panel B: RNA-EMSA experiment using complete HeLa nuclear extract and the RNA probes of Panel A. The different radioactive RNA probes were incubated with 2 µg of HeLa nuclear extract and 15 µg/µl of heparin to generate more stringent incubation conditions. The experiment was performed in more stringent conditions (see Materials and Methods section for details) than those used in Figure 5 to make more evident the differences in complex formation efficiency.

When we performed the EMSA experiments in more stringent conditions, we observed that the ΔDSE probe was not able to form stable complexes when incubated with HeLa nuclear extracts (Figure 6B, Lane 4), supporting the cell transfection experiments’ results shown above (Figures 2B), and the binding and competition experiments shown in Figure 5. Deletion or mutation of the USE resulted in an increase formation of RNA-protein complexes (Figure 6B, Lanes 3 and 5), as already observed above using less stringent conditions (Figure 5, Lane 8). Mutation of the DSE also resulted in a decrease in the formation of RNA-protein complexes (Figure 6B, Lane 6).

**Figure 7:**
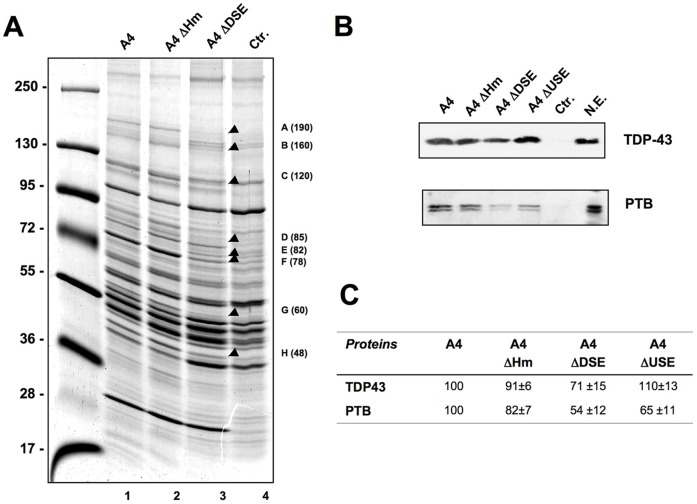
Identification of proteins bound to the A4 PAS by pull down analysis. **Panel A:** Pull down experiment of HeLa nuclear proteins incubated with the different RNA probes. Bound proteins were separated by SDS-PAGE and stained with colloidal Coomassie blue. The arrows indicate differentially bound proteins (label and apparent size), which were purified and identified by mass spec analysis (results are listed in Table 1). Molecular weight markers are indicated on the left. **Panel B:** Western blot analysis of the pulled down proteins TDP-43 and PTB. “Ctr” and “NE” indicates control beads and input nuclear extract, respectively. **Panel C:** Quantification of the results shown in Panel B (three independent experiments, mean±SD).

To identify the proteins participating in Add2 polyadenylation at the A4 PAS, we implemented a pull-down approach using the same RNAs probes and HeLa nuclear protein extract used above. Proteins were allowed to bind to the different versions of the A4 PAS. Bound proteins were eluted and separated in a SDS-PAGE (Figure 7A). Surprisingly, no significant differences in the levels of pulled-down proteins by the wt A4 control RNA and the ΔH RNA were observed (Figure 7A). When we performed the pull down analysis using the ΔUSE RNA the only evident difference observed was a reduction in the levels of a ∼60 kDa band that was later identified as PTB (data not shown). On the contrary, several pulled-down bands showed reduced levels when ΔDSE RNA was used (indicated by arrows and letters in Figure 7A). We purified these bands and identified them by mass-spectrometry analysis. The results of the analysis are listed in Table 1. We then performed Western blot analysis of the pulled-down proteins to confirm the changes in the levels of TDP-43 and PTB, well-known RNA binding proteins participating in pre-mRNA processing (Figures 7B and 7C). We observed a decrease in TDP-43 signal for the ΔDSE pulled-down proteins, while a decrease of PTB levels was observed when the ΔDSE and ΔUSE RNAs were used, confirming the differences observed in the Coomassie-blue staining of the pulled-down proteins (Figure 7A).

**Table 1:** Mass spectrometry results.

Bands (relative size)	Proteins	Relative levels
A (∼190 kDa)	Vigilin	Reduction
B (∼160 kDa)	RNA Helicase A	Reduction
C (∼120 kDa)	Nucleolin	Reduction
D (∼85 kDa)	FBP2	Absence
E (∼82 kDa)	Septin	Increase
F (∼78 kDa)	FBP1	Reduction
G (∼60 kDa)	PTB1	Reduction
H (∼48 kDa)	TDP-43	Reduction

The bands purified in Figure 7A were identified by mass spectrometry. The relative levels observed in the ΔDSE pull down respect to the wt A4 RNA are indicated.

## Discussion

In the present report we used chimeric minigenes to study the mechanisms of polyadenylation of the mouse Add2 gene. We accurately reproduced cleavage and polyadenylation at the distal PAS of the Add2 pre-mRNA. We have shown that the Hm and DSE promote efficient use of this polyadenylation signal, as their deletion or point mutation resulted in the activation of cryptic PASs. This region actively interacts with nuclear proteins. We have identified some of the putative proteins recognizing the USE and DSE of Add2 pre-mRNA. Among them we found proteins known to participate in RNA metabolism and processing, such as PTB, TDP-43, FBP1, FBP2 and RNA Helicase A.

Many different Hm variants are present in genes, being the “classical” AAUAAA together with the AUUAAA variant the most common versions, found in about 70–80% of genes [32], [33]. In the case of the Add2 A4 PAS, the Hm is AGUAAA, which is an Hm variant present in about 3.7% of PAS [32], [33]. The occurrence of less frequent (or somehow weaker) Hm is usually associated to the presence of a stronger DSE [13]. In the case of the Add2 A4 PAS, reversion of the AGUAAA to the canonical AAUAAA resulted in an increase in the amount of Add2 chimeric mRNA (Figure 4). This reasoning, together with our experimental data, led us to focus our attention on the DSE. In fact, we have shown that deletion or mutation of the Add2 DSE, both in the presence of the less active natural AGUAAA or the more active canonical AAUAAA hexanucleotide motifs, resulted in the activation of cryptic PASs located ∼200 bases upstream of the natural PAS, underscoring the importance of the DSE in the definition of the Add2 A4 PAS. The importance of the DSE in the distal Add2 PAS definition is also supported by the RNA-EMSA experiments, in which an RNA probe lacking the DSE formed weaker RNA-protein complexes and was not able to compete the RNA-protein complexes formed by the wt RNA probe. In addition, the activation of these cryptic sites nearby the natural A4 PAS suggests the presence of other accessory elements in the A4 PAS region. This hypothesis is supported by the fact that both the cryptic PASs and the potential cryptic core elements are located within a highly conserved region, which spans for about 550 bases, starting about 525 bases upstream of the CS and ending 22 bases downstream of the CS (Figure S1B). This peculiarity suggests that a selective pressure was acting to maintain a specific function, being the only highly conserved region in the unusually long Add2 3′UTR.

We observed that deletion or point mutation of the A4 PAS USE results in an increase in the amounts of mRNA suggesting that this region negatively regulates mRNA processing or stability. However, we did not observe any difference in the pull down analysis respect to the wt probe. Bioinformatic search of seed sites for miRNAs overlapping with the USE showed that miRNAs could potentially bind that region. Some of these miRNAs are expressed in HeLa and HEK293 cells with, thus, the potentiality to bind the Add2 3′ UTR. However, only the experimental validation by point mutation of the binding site together with miRNA depletion or overexpression will tell us whether the increase observed after USE deletion could be related to the elimination/mutation of a putative miRNA binding site.

Add2 pre-mRNA undergoes tissue-specific regulation of APA. Erythroid tissues use proximal PASs while a distal PAS is preferentially used in brain tissues [17]. This polyadenylation pattern is also observed in many other genes, where proximal PASs are used in actively dividing tissues, while distal PASs are preferentially used in highly differentiated tissues [34], [35], [36]. Longer variants of the 3′UTR are thought to reduce protein output, probably by affecting mRNA stability and/or translation efficiency [36]. Consequently, the use of alternative distal PAS has important consequences in the fate of mRNAs. Despite the important regulatory effects that APA has on gene expression, the mechanisms leading to the use of alternative polyadenylation sites are still not completely understood. A few examples have been proposed and mechanistically characterized so far [37], [38], [39], [40], [41], [42]. However, these mechanisms do not explain all cases of APA. In fact, changes in Add2 mRNA polyadenylation were not detected in any of the published high throughput screenings [40], [41], [42] suggesting that other mechanisms acting in the selection of alternative PAS need yet to be determined.

As mentioned above, we focused our attention on the proteins that bind to the distal Add2 A4 PAS and may have a potential role in 3′ end processing. Surprisingly, among the proteins identified in the pull down experiments we did not detect any protein of the core polyadenylation machinery, such as CPSF-160 and CstF-64, known to bind to the hexanucleotide motif and DSE, respectively. Among the identified bands we found Polypyrimidine Tract Binding Protein (PTB), a well-known RNA binding protein in the splicing field that preferentially binds to UCUU(C) and UUCU/C sequences in a pyrimidine-rich context [43]. PTB binds the polypyrimidine tract of 3′ splice sites and its primary role is in the regulation of alternative splicing via exon silencing [43]. Moreover, it has been reported that PTB participates in polyadenylation by interacting with the U/Py-rich USE of COX-2, C2, GFAP and alpha-TM mRNAs [7], [44], [45], [46]. PTB plays a positive role in pre-mRNA 3′ end processing, in vitro promoting 3′ end cleavage and polyadenylation, and recruiting hnRNP H to G-rich sequences associated with several pA signals [47]. In another model, overexpression of PTB results in a 75% reduction of mRNA levels due to reduced efficiency of 3′ end cleavage, due to competition with CstF for recognition of the pA signal’s pyrymidine-rich DSE [4]. PTB also regulates CT/CGRP through the interaction with the poly(A) site to positively affect polyadenylation to produce CT [48]. Despite the fact that consensus PTB binding sites are not evident in the USE or DSE of Add2, a significant change in PTB levels was detected in the pulled-down proteins (Figure 7 and Table 1) suggesting that PTB could also have a role in Add2 polyadenylation.

Another interesting protein detected in the pull-down analysis was TAR-DNA binding protein-43 (TDP-43), a protein with DNA and RNA binding properties. Reported TDP-43 nuclear functions are multiple, ranging from the regulation of HIV transcription [49], alternative splicing [50], miRNA biogenesis [51] and TDP-43 mRNA levels autoregulation [52]. It was shown that many of these functions occur through TDP-43 binding to a stretch of at least four GU repeats, the most common binding site of TDP-43 [50], [53]. The Add2 DSE contains a stretch of four UG repeats immediately downstream of the CS, which is also a putative binding site for CstF64. We have shown that these repeats are active in polyadenylation, since their mutation results in a reduction of Add2 mRNA levels and activation of cryptic PASs, and we hypothesize that TDP-43 may, directly or indirectly, possibly through the competition or interaction with CstF64, participate in Add2 polyadenylation. A regulatory mechanisms based on the competition between TDP-43 and CstF64 was recently shown for the autoregulation TDP-43 mRNA levels [52], [54], and a similar mechanisms could be acting in the Add2 DSE. TDP-43 was also found as a binder of the USE of the prothrombin PAS, an U-rich element having only two UG repeats, although it doesn’t seem to have a prominent role in polyadenylation of this pre-mRNA [55]. As expected, TDP-43 was not found among the components of the pre-mRNA 3′ processing complex of RNA substrates that do not contain GU repeats in their DSE, as the SV40L and adenovirus L3 pre-mRNAs [56]. The potential involvement of TDP-43 in polyadenylation is supported by the results of Freibaum et al, who showed that TDP-43 interacts with members of the polyadenylation and splicing machinery [57]. The possible involvement of TDP-43 in polyadenylation may have major significance in the understanding of the mechanisms leading to some disease states. In fact, TDP-43 is mislocalized from its normal nuclear localization to the cytoplasm of diseased motor neurons of ALS and FTLD patients, and nuclear depletion of TDP-43 may have important consequences in mRNA metabolism.

FAR-upstream Binding protein 1 (FBP1), originally identified as a DNA binding protein regulating c-Myc gene transcription, was shown in recent years to act as an RNA-binding protein and to regulate mRNA translation or stability of genes by binding to A/U rich sequences (ARE), competing with members of the HuR family [58], [59]. FBP2/KSRP is a multifunctional RNA binding protein showing extensive aminoacid sequence identity with FBP1, that modulates many steps of RNA life including pre-mRNA splicing, ARE-mediated mRNA decay, and maturation of selected miRNAs from precursors [60], [61]. It was recently shown that FBP2 binds defined USE identified in a number of genes, like prothrombin, inhibiting the 3′end processing [62], supporting the hypothesis of a possible role of FBP2 in Add2 pre-mRNA polyadenylation.

All of the other identified proteins, except Septin-9 [63], [64], were shown to directly participate in mRNA metabolism, but their involvement in polyadenylation has not been reported so far. Nucleolin is another multifunctional protein capable of interacting with DNA and RNA. Nucleolin has been reported to interact with the 3′ UTR of numerous mRNAs, enhancing their stability and translability [65], although its main function is in the nucleolus, where it interacts with precursor ribosomal RNA and is essential for its biogenesis and transport to the cytoplasm [66]. Vigilin is a protein containing multi hnRNP K homology domains able to bind RNA that participates in the regulation of mRNA stability. It also interacts with tRNA and, in association with other factors as a multi-protein complex, contributes to tRNA export from the nucleus [67]. Moreover, it binds in vitro the vitellogenin mRNA 3′UTR preventing its endonuclease cleavage [68]. Vigilin also interacts with *c-fms* mRNA 3′UTR destabilizing the transcript and negatively regulating its translation [69]. RNA helicase A, also denominated DHX9, is another RNA-binding protein with ATPase and RNA helicase activities. It also participates mRNA metabolism by activating several transcriptional pathways [70] and promoting translation of selected messenger RNA containing defined elements in the 5′UTR [71], [72].

To summarize, we have characterized the core polyadenylation elements of the distal PAS of Add2. We have shown that nuclear proteins bind the A4 PAS RNA, and that the formation of protein-RNA complexes depends on the presence of the DSE. We identified some of the proteins that bind to the DSE element and among them we found proteins known to participate in polyadenylation, such as PTB and FBP2. We also found proteins reported to be involved in splicing and stability of mRNAs, such as PTB-43, vigilin, FBP1 and FBP2, nucleolin. Future experiments will be necessary to determine their functional role in Add2 polyadenylation.

## Supporting Information

Figure S1 **Alignment of the distal PAS of the Add2 gene (A4 PAS). Panel A:** The sequence of the distal A4 PAS of the Add2 gene is shown (234 bp), together with the reported ESTs for that region (from the UCSC browser). The USE, Hm, cleavage site and DSE are indicated (red and orange rectangles). The homology among vertebrates, mammals and Euarchontoglires is shown (bottom). **Panel B:** A lower magnification scheme of the same region is shown, corresponding to 869 bp.
(TIF)

Figure S2 **Alignment of the proximal PAS of the Add2 gene (A1 PAS):** Scheme showing the A1 PAS and the other annotated transcripts for that region. The USE, Hm, cleavage site and DSE are indicated (red and orange rectangles). The homology among vertebrates, mammals and Euarchontoglires is shown (bottom).
(TIF)

Figure S3 **Alignment of the A23 PAS of the Add2 gene:** The scheme shows the annotated transcripts in other species for that region. The canonical polyadenylation elements were not detected, as they highly differ from the consensus ones. The homology among vertebrates, mammals and Euarchontoglires is shown (bottom).
(TIF)

Figure S4 **Sequence of the A4 PAS region in the deleted and mutated constructs:** The scheme shows the sequence of the A4 PAS region of the RNAs used for the cell-transfection experiments, and for the band shift and pull down analysis. The mutations introduced are indicated in red (underlined). The USE, hexanucleotide motif and DSE are indicated.
(TIF)
