# Supplementary figures and images for: Characterization of the Distal Polyadenylation Site of the ß-Adducin (Add2) Pre-mRNA
Source: PLoS One. 2013 Mar 15;8(3):e58879. doi: 10.1371/journal.pone.0058879 (PMC3598803; doi:10.1371/journal.pone.0058879)

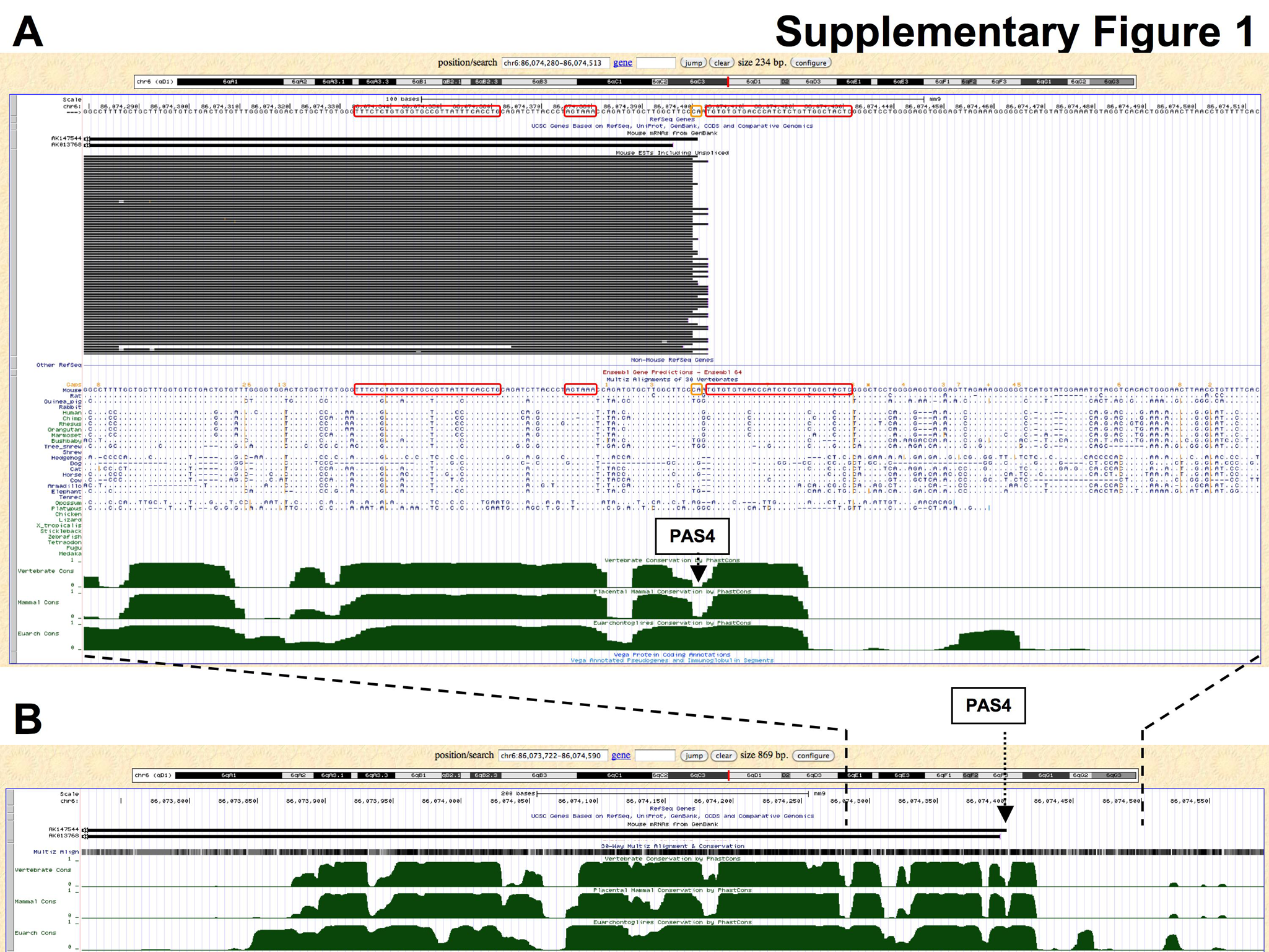

Supplement: Figure S1 — Alignment of the distal PAS of the Add2 gene (A4 PAS). Panel A: The sequence of the distal A4 PAS of the Add2 gene is shown (234 bp), together with the reported ESTs for that region (from the UCSC browser). The USE, Hm, cleavage site and DSE are indicated (red and orange rectangles). The homology among vertebrates, mammals and Euarchontoglires is shown (bottom). Panel B: A lower magnification scheme of the same region is shown, corresponding to 869 bp. (TIF) [file pone.0058879.s001.tif]

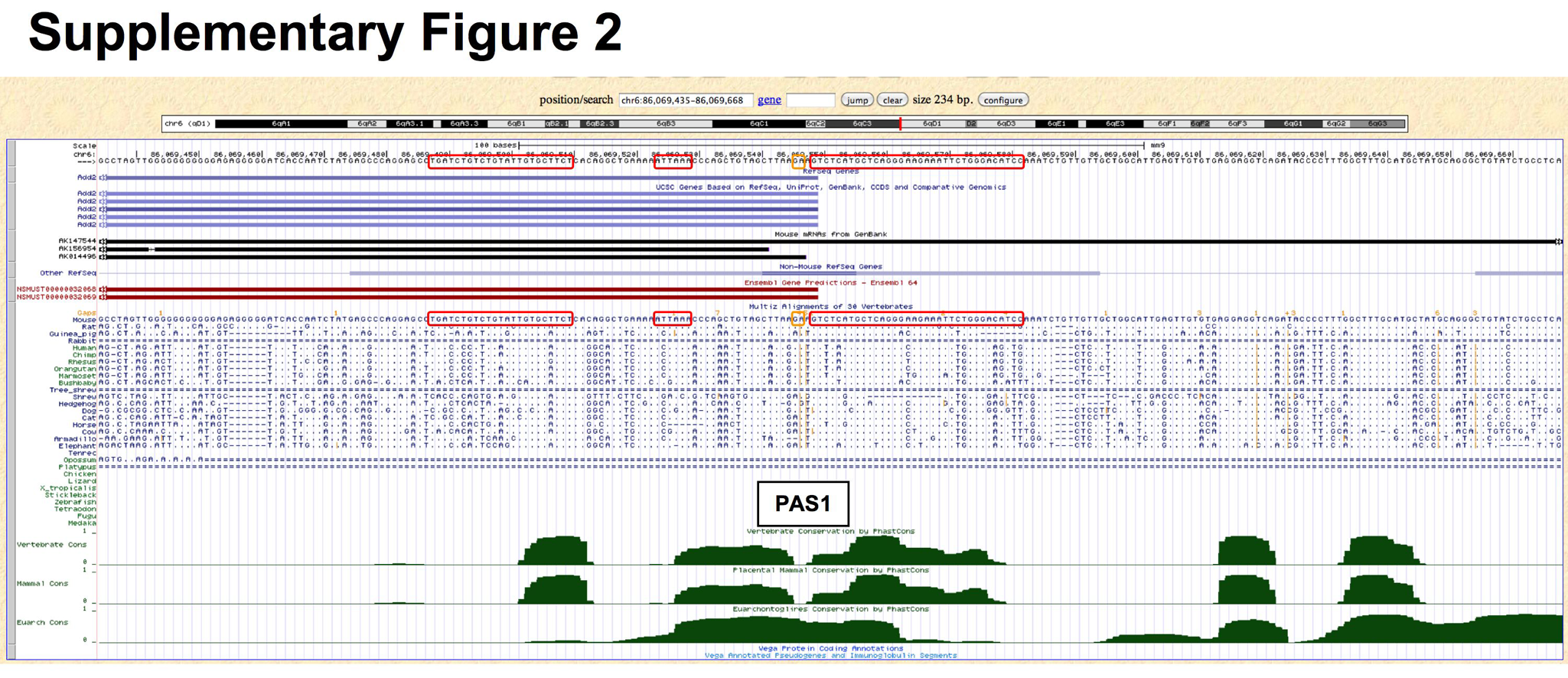

Supplement: Figure S2 — Alignment of the proximal PAS of the Add2 gene (A1 PAS): Scheme showing the A1 PAS and the other annotated transcripts for that region. The USE, Hm, cleavage site and DSE are indicated (red and orange rectangles). The homology among vertebrates, mammals and Euarchontoglires is shown (bottom). (TIF) [file pone.0058879.s002.tif]

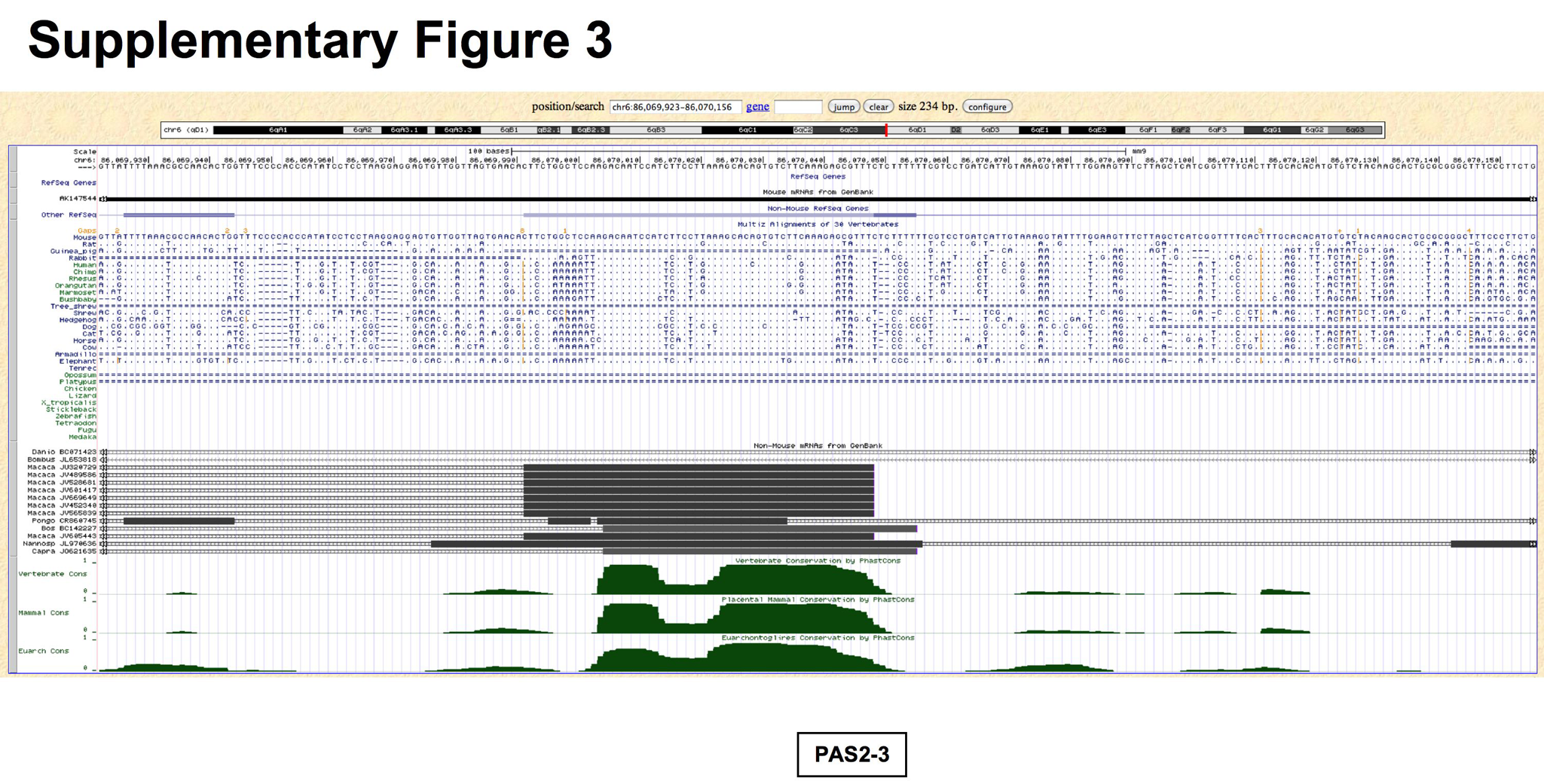

Supplement: Figure S3 — Alignment of the A23 PAS of the Add2 gene: The scheme shows the annotated transcripts in other species for that region. The canonical polyadenylation elements were not detected, as they highly differ from the consensus ones. The homology among vertebrates, mammals and Euarchontoglires is shown (bottom). (TIF) [file pone.0058879.s003.tif]

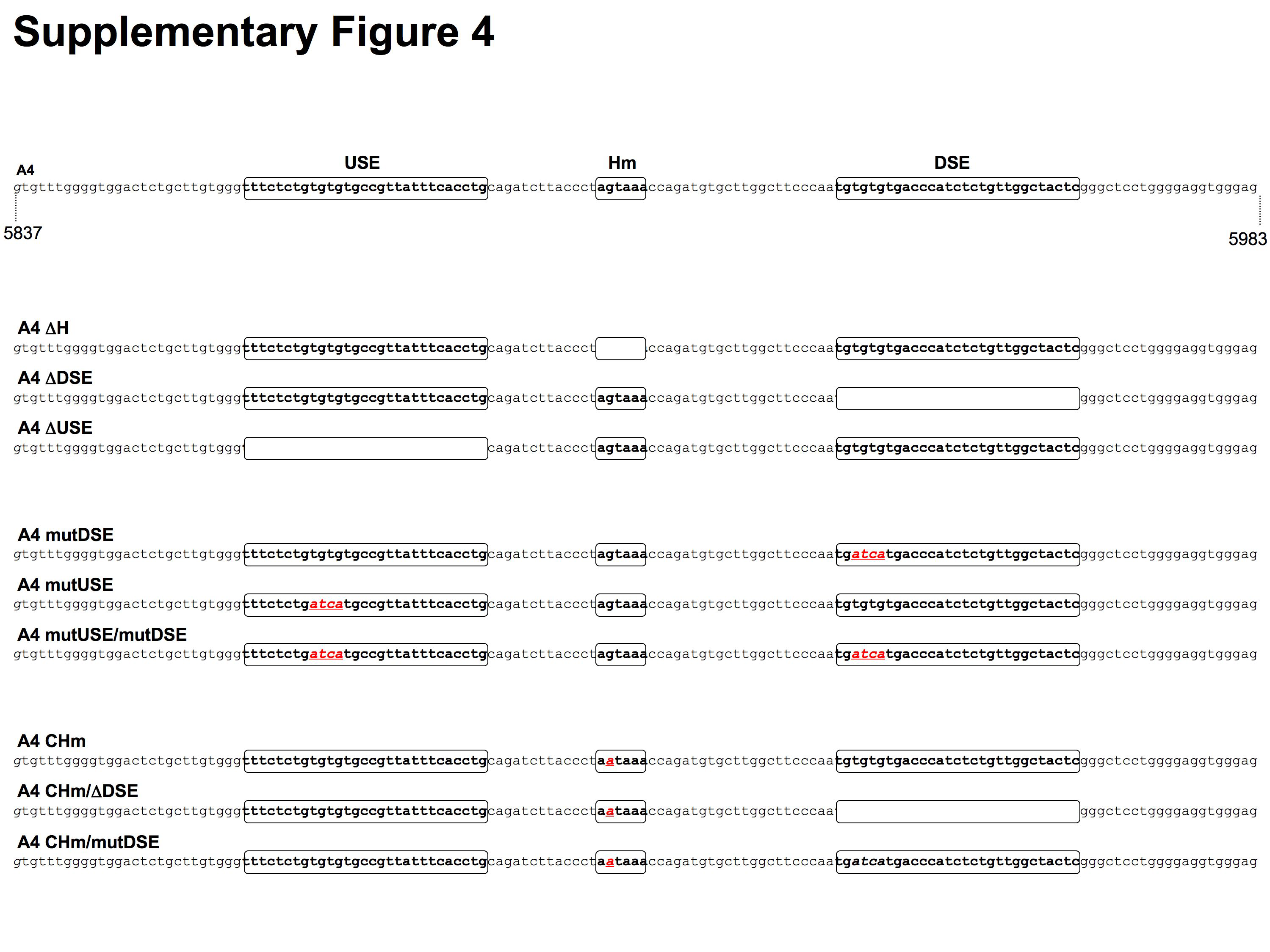

Supplement: Figure S4 — Sequence of the A4 PAS region in the deleted and mutated constructs: The scheme shows the sequence of the A4 PAS region of the RNAs used for the cell-transfection experiments, and for the band shift and pull down analysis. The mutations introduced are indicated in red (underlined). The USE, hexanucleotide motif and DSE are indicated. (TIF) [file pone.0058879.s004.tif]
